# Blue Light in Dermatology

**DOI:** 10.3390/life11070670

**Published:** 2021-07-08

**Authors:** Magdalena Sadowska, Joanna Narbutt, Aleksandra Lesiak

**Affiliations:** Department of Dermatology, Pediatric Dermatology and Dermatological Oncology, Medical University of Łódź, 90-419 Łódź, Poland; joanna.narbutt@umed.lodz.pl (J.N.); aleksandra.lesiak@umed.lodz.pl (A.L.)

**Keywords:** blue light, phototherapy, dermatology

## Abstract

Phototherapy is an important method of dermatological treatments. Ultraviolet (280–400 nm) therapy is of great importance; however, there are concerns of its long-term use, as it can lead to skin aging and carcinogenesis. This review aims to evaluate the role and the mechanism of action of blue light (400–500 nm), a UV-free method. The main mediators of cellular responses to blue light are nitric oxide (NO) and reactive oxygen species (ROS). However, the detailed mechanism is still not fully understood. It was demonstrated that blue light induces an anti-inflammatory and antiproliferative effect; thus, it may be beneficial for hyperproliferative and chronic inflammatory skin diseases such as atopic dermatitis, eczema, and psoriasis. It was also found that blue light might cause the reduction of itching. It may be beneficial on hair growth and may be used in the treatment of acne vulgaris by reducing follicular colonization of *Propionibacterium acnes*. Further studies are needed to develop accurate protocols, as the clinical effects depend on the light parameters as well as the treatment length. There are no major adverse effects observed yet, but long-term safety should be monitored as there are no studies considering the long-term effects of blue light on the skin.

## 1. Introduction

Phototherapy is often used in the management of many common skin diseases. Its effect depends on wavelength, frequency, and the mechanism of action of light, but also on the irradiation time and the dose. The radiation spectrum includes infrared radiation (IR, 760–1000 nm), visible light (400–760 nm), and ultraviolet radiation (UV, 280–400 nm) [1,2]. The mechanism of action of visible light is not as fully understood as the widely used UVB. In the visible light spectrum there is red, orange, yellow, green, blue, and violet light. In this review we focus on better understanding of the role of blue light in dermatology. In recent years, the UV-free blue light phototherapy method (400–500 nm) has been attracting more attention. Despite the many advantages of UV treatment, there are some concerns of its long-term use, as it can lead to carcinogenesis and skin aging [3,4,5,6,7]. Therefore, there is a need to look for alternative, safer methods.

## 2. The Mechanism of Action of Blue Light

UVB interacts with cells of epidermis, whereas UVA reaches deeper layers of the skin and affects immune cells of epidermis and dermis [8]. Comparing with UV, visible light acts deeper in the dermis, but at the same time more superficially than infrared radiation. Hemoglobin and melanin of the epidermis are highly absorbing the visible light. The maximum penetration of blue light is 0.07–1 mm [1,9]. Chromophores are the molecules that absorb light, such as in the following in the skin: endogenous nucleic acids, aromatic amino acids, urocanic acid, tryptophan, tyrosine, NADPH, NADH cofactors, cytochromes, riboflavins, porphyrins, melanin and melanin precursors, protoporphyrin IX, bilirubin, hemoglobin, β-carotene, or water molecules [1]. Therefore, the effect of blue light is dependent on different chromophores (photoacceptors). The main and the most important photoacceptors are opsins, flavins, porphyrins, and nitrosated proteins (e.g., S-nitro-albimin) [2].

It is suggested that blue light may affect mitochondrial function through cytochrome c oxidase, which is the complex IV of the electron transport chain, found in the mitochondrial membrane [10,11]. Dungel et al. demonstrated that blue light at the wavelength of 430 nm reactivates the mitochondrial respiratory function after inhibition with NO [10].

The role of opsin (OPN) (which are G-protein receptors) is also investigated as they are activated by blue light. Depending on location of their expression, there are different categories of opsins. OPN2, OPN3, and OPN4 are expressed in the epidermis [11]. The opsin receptor is possibly excited by blue light, stimulating the transient receptor potential channels and then causing a flood of calcium, which triggers calcium/calmodulin-dependent protein kinase-II (CAMKII) and in the end causes gene transcription changes [11]. OPN2 (Rhodopsin) and OPN3 (Panopsin, Encephalopsin) were found to be expressed not only in the skin, but also in the anagen hair follicle. Buscone et al. demonstrated that irradiation with blue light (3.2 J/cm**^2^**, 453 nm) caused elongation of anagen phase in hair follicles ex vivo [12]. Opsin’s role has also been investigated in the modulation of pigmentation and melanogenesis, but only in the Fitzpatrick skin type III and higher. It was found that blue light affects melanocytes directly and through OPN3 impacts the melanogenesis, which is calcium dependent. Blue light causes production of multimeric tyrosinase, which results in tyrosinase stimulation in melanocytes of the higher Fitzpatrick phototype [13].

Another potential mechanism of blue light includes the activation of flavins and flavoproteins. Flavin mononucleotide (FMN) and flavin adenine dinucleotide (FAD) exposed to irradiation increase reactive oxygen species (ROS) formation twofold [14]. In various cells, proteins that contains flavins can be found [2]. One of them is cryptochromes [15]. In a recent study, Buscone et al. suggested that blue light via cryptochrome 1 (CRY1) may induce a positive effect on hair growth, as it locates in the hair follicle after irradiation with 453 nm. In ex vivo hair follicles, prolongation of the anagen phase was seen, which might be connected to the increase in the level of CRY1 during exposure to blue light [15].

**Figure 1:**
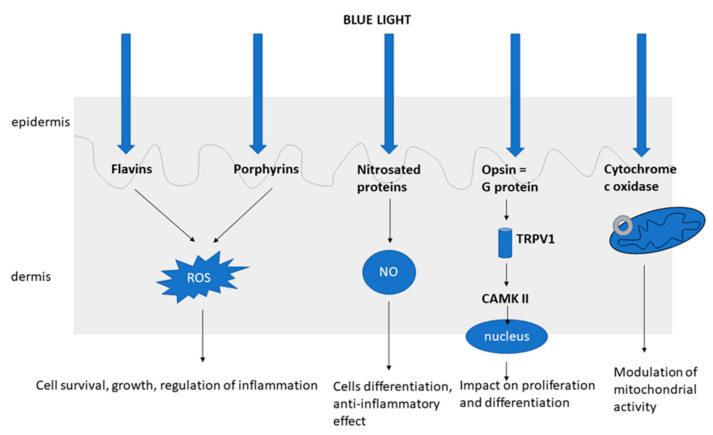
The mechanism of action of blue light [2,10,11,14,16,17,18,19,20,21].

Other blue light photoacceptors are porphyrins, which are heterocyclic aromatic compounds. Enzymes that contain porphyrin are present in various cells, such as hemoglobin, cytochrome p-450 enzymes, and the complexes of the electron transport chain [2,11]. It is suggested that irradiation with blue light by excitation of porphyrins leads to ROS formation [2,16,17,18] (Figure 1).

## 3. Antiproliferative and the Anti-Inflammatory Properties of Blue Light

Becker et al. investigated the effect of blue light on keratinocytes and showed a reduction in the proliferation of these cells. In addition, increased oxidative stress was observed, which can be explained by the increased production of ROS in response to blue light irradiation. Physiologically reactive oxygen species are mainly produced in mitochondria in complex I and III of the electron transport chain [22]. The described effect depended on the exposure time, and a reduction in the proliferation of keratinocytes was observed after 15 min of irradiation. The study revealed an increase in the transcription of electron transport chain genes, cytochrome P450 genes, and steroid hormone genes. The authors, on the other hand, reported decreased expression of inflammation genes and suggested this was due to stimulation of steroid hormone production through the CYP pathway, which has anti-inflammatory effects [23].

Another explanation for decreased proliferation may be nitric oxide (NO)-mediated initiation of differentiation of keratinocytes [19]. Oplander et al. demonstrated that blue light (λ = 420 and λ = 453 nm) triggered NO production from S-nitroalbumin and aqueous nitrite solutions, but also resulted in an increase in free NO in the dermis in vivo [20]. Similarly, Mittermayr et al. observed the ability of blue light to detach NO from NO-hemoglobin compounds [24]. Yoo et al. found that blue light (λ = 470–480 nm, 76 W/m^2^) reduced the proliferation of human keratinocytes. Moreover, the authors observed a rise in the amount of TRPV1, which is one of the transient receptor potential (TRP) cation channels that is also observed in keratinocytes. It affects not only various signaling pathways, but also increases the level of pro-inflammatory cytokines and differentiation, and reduces proliferation. The authors demonstrated that the increase of TRPV1 caused EGFR destruction, which led to the inhibition of AKT/GSK3β/FoxO3a signaling, and in the end to the decrease of keratinocytes proliferation [25]. Thus, the main theory is that blue light via chromophores has an impact on proliferation and differentiation [23].

Various mechanisms are involved in ROS signaling. One of them is a Nrf2-dependent mechanism, which is a “basic leucine zipper protein” involved in the expression of antioxidant factors. By inhibiting NF-kB, which regulates the pro-inflammatory response, Nrf2 has an anti-inflammatory effect [11,26]. In the studies analyzing the influence of blue light on Nrf2 signaling, Trotter et al. observed upregulation of Nrf2 expression in vitro. In addition, the authors showed an effect on the inflammatory response of the human monocyte THP-1 cell line in response to blue light irradiation. Blue light reversed the cytotoxic effect of LPS (lipopolysaccharide), and irradiations decreased cytokine generation in response to 0.1 μg/mL LPS, but this effect was not observed at high LPS levels [26]. In the previous studies it was found that blue light treatment induced upregulation of Nrf2 in A431 epidermoid carcinoma cells and resulted in significantly increased levels of heme oxygenase 1 (HO-1), an anti-inflammatory and antioxidative factor [27]. These results may support the theory of the anti-inflammatory properties of blue light. Thus, it can be a therapeutic method in chronic inflammatory skin diseases.

The anti-inflammatory properties of blue light were also observed in other studies. Blue light irradiation (λ = 400–500 nm, at the intensities 3.75, 7.5, or 15 J/cm^2^, total fluence 43.7 J/cm^2^) of dendritic cells (DC) in vitro did not cause cell degradation. It reduced DC activation and maturation and decreased their effect on cytokine secretion by T cells, which produced a reduced amount of IFN-γ, IL-2, IL-10, IL-12p70, IL- 1β, and TNF-α (apart from IL-4) with the most effective results at higher doses. This indicates that the blue light has an anti-inflammatory effect [28]. Other findings have shown that blue light irradiations of monocyte-derived dendritic cells precursors (MDDCp) had no effect on monocyte-derived dendritic cell immature phenotype (iDCs) and did not affect the growth of mature dendritic cells. Thus, blue light did not affect differentiation and maturation of dendritic cells. However, the authors observed lower IL-6 and TNF-a generation by MDDCs dose dependently [29]. Kim et al. showed that the anti-inflammatory effect of blue light is NO and S-nitrosylation dependent and not by ROS [21]. Thus, blue light may be used for the treatment of hyperproliferative disorders and chronic inflammatory diseases.

## 4. The Negative Aspects of Blue Light

J. Liebmann et al. found that blue light at 453 nm does not have a negative effect on human skin cells (keratinocytes and endothelial cells) up to a fluence of 500 J/cm^2^ [19]. Similarly, Oplander et al. showed that blue light was nontoxic to human fibroblasts at 453 nm, but also at 480 nm. However, blue light at 410 and 420 nm caused increased oxidative stress as well as being toxic depending on the dose and wavelength. Moreover, small doses of blue light (λ = 410, 420, 453 nm) decreased the antioxidative properties of fibroblasts. The authors demonstrated that fibroblasts may be more sensitive to blue light as a decrease in proliferation was observed after irradiations at 410, 420, and 453 nm at not toxic doses [30]. 

There are some studies showing the negative effect of blue light. Dong et al. observed that blue light at 410 nm caused a reduction in PER1 transcription in keratinocytes. It is a clock gene, involved in the circadian rhythm, which may suggest that skin cells are able to control the clock gene expression depending on the light sensation. The authors suggested that blue light may disrupt the nighttime rhythm of skin cells, which is important for the regeneration and repair. It can make cells feel like it is daytime at night. The PER1 level was reduced after 3 h in comparison to cells protected from the light. Additionally, they showed an increase in ROS production, DNA damage by 53%, and inflammatory mediator production (IL-1α, IL-6, IL-8, and TNF-α) after blue light irradiation. Exposure of human keratinocytes to 200 J/cm^2^ (66 min) resulted in a 147% increase of ROS generation. Authors suggested that exposure to blue light may potentially cause skin damage and skin aging [31]. Similarly, Yoo et al. demonstrated that blue light caused a rise of ROS release, which was dependent on transient receptor potential vanilloid 1 (TRPV1). They also found that blue light increased proinflammatory cytokine TNF-α production by activating of activator protein 1 (AP-1) and nuclear factor κB (NF-κB) [25]. In the study investigating the ROS formation following phototherapy, Nakashima et al. found that blue light caused oxidative stress in human keratinocytes. The effect was greater after UVA irradiations than the blue light. Blue light through flavin excitation caused production of ROS, probably superoxide, which may promote skin aging, whereas UVA induced formation of singlet oxygen [32]. Vandersee et al. observed that blue-violet light irradiation of the skin (80% in 380–495 nm, maxima 440 nm, 100 mW/cm^2^) induced destruction of the carotenoids dose dependently. These antioxidants were reduced by 13.5% after irradiation at 50 J/cm^2^ and 21.2% after irradiation at 100 J/cm^2^. Their levels returned to the original level after 1 h for the dose of 50 J/cm^2^ and 24 h for the dose of 100 J/cm^2^. The authors suggested that the destruction of dermal carotenoids indicates the number of produced free radicals, mainly ROS in the skin [33].

Liebmann et al. found that blue light (λ = 412, λ = 419, and λ = 426 nm at high fluences 66–100 J/cm^2^ and λ = 453 nm at very high fluences >500 J/cm^2^) is toxic for endothelial cells and keratinocytes. After three consecutive days of irradiation every 24 h, the amount of endothelial cells and keratinocytes were decreased in cultures irradiated with 412, 419, or 426 nm compared to the nonirradiated cells. This was dependent on the dose as well as the wavelength. On the other hand, blue light at 453 nm up to fluence of 500 J/cm^2^ was not toxic [19]. However, in the recent study it was reported that in keratinocytes, no apoptosis occurred 24 h after 30 min irradiation with blue light (λ = 453 nm, 23 mW/cm^2^). The authors investigated the impact of blue light on ROS release, apoptosis, and gene expression after irradiation of human keratinocytes in vitro. A rise in ROS amount was observed 30 min after irradiation, which returned to its original levels already 1 h after irradiation. Moreover, the induction of ROS production did not destroy the cells, and apoptosis was not detected. Analyzing gene expression after blue light irradiation, its effect could be seen already after 1 h. The authors discovered the aryl hydrocarbon receptor, which could be a target for blue light, as at certain doses it might act in a cell protective manner, accompanied with reduced proliferation, production of steroid hormones, and inhibition of inflammatory responses. It is possible that the mechanism responsible for AHR activation may be the photo-oxidation of tryptophan by blue light [34].

Therefore, blue light at 453 nm using an intensity of 23 mW/cm^2^ and up to fluence of 500 J/cm^2^ does not cause apoptosis of keratinocytes, whereas T cells are susceptible to apoptosis at lower energy levels, with the fluences that are not toxic for keratinocytes and endothelial cells [19,34]. While small amounts of ROS may protect the cells, larger amounts of ROS can damage them [35]. However, there is a theory that the viability of cells depends on their mechanisms to deal with oxidative stress [30]. Wataha et al. found that blue light modulates cell survival and growth in different ways, and it depends on how much energy the cells use. Rapidly dividing cells, requiring more energy, were more sensitive to the inhibitory effects of blue light, but it is still unknown how exactly [36]. 

In the case of UV irradiation, there are confirmed long-term side effects such as skin aging and carcinogenesis [3,4,5,6,7]. Considering the risks of UV treatments, blue light seems to be a safer therapeutic option. However, as it is an innovative method, long-term safety should be monitored.

## 5. Effect on Pigmentation

Duteil et al. analyzed the influence of blue (λ = 415 nm) and red light (λ = 630 nm) on the increase in pigmentation in vivo and found correlation with blue light spectrum in patients with III and IV skin phototype. Moreover, the hyperpigmentation persisted during the 3-month follow-up and was more intense compared to patients exposed to UVB. This effect was not observed considering the red light. However, the mechanism behind this phenomenon is unknown [37]. Hyperpigmentation was also observed in other clinical studies [38,39,40]. In patients with psoriasis vulgaris treated with blue light (λ = 420 nm and 453 nm), the only side effect observed was hyperpigmentation in 50% of the patients, which was not permanent and it disappeared after treatment was finished [39]. In another study of patients with psoriasis, hyperpigmentation was reported in 80% of subjects after blue light irradiation [40]. Kleinpenning et al. analyzed skin biopsies of patients with no skin lesions after 5 days of blue light irradiations (Waldmann 450L photodynamic therapy lamp, 20 J/cm^2^ two times a day) and a mild hyperpigmentation was noted, confirmed by Melan-A-positive cells found in the skin exposed to blue light. However, no impact on DNA damage of skin cells, early photoaging, and inflammatory cell infiltration was seen [41].

## 6. Anticancer Effect 

There are some studies in vitro that demonstrated the anticancer effect of blue light. Ohara et al. demonstrated that blue light (λ = 470 nm, irradiance 5.7 mW/cm^2^) inhibited the growth of B16 melanoma cells, which was related to the irradiation time; however, no rise of apoptosis was observed. The number of G0/G1- and G2/M-phase cells was higher and the number of S-phase cells was lower after irradiations, although the exact mechanism is not known [42]. In the next studies, Ohara et al. showed that adding riboflavin to the B16 melanoma cells exposed to blue light (λ = 470 nm, irradiance 5.7 mW/cm^2^) induced cell damage. This effect was not observed after irradiation with green or red light, indicating the influence of ROS was generated due to the absorption of blue light by riboflavin [43]. Thus, combining blue light with photosensitizing factors may increase the effectiveness of photodynamic therapy (PDT). Similarly, Sparsa et al. found that irradiation with blue light of B16F10 melanoma and bovine endothelial cells (λ = 450 nm, 10 J/cm^2^) caused cell necrosis. Suspecting that this phenomenon could be triggered by ROS production, the authors monitored the oxidative stress of the cells. However, no effect was observed at 10 J/cm^2^ or 20 J/cm^2^, suggesting that a different mechanism was responsible for cell damage [44]. In the recent study, Chen et al. showed the suppressive effect of blue light (λ = 418 nm and 457 nm) on the growth of melanoma. The wavelength 457 nm was more effective in inhibiting the growth and migration of B16F10 melanoma cells. Since in OPN3, one of the photoreceptors absorbs blue light with a wavelength of 460 nm, it is suspected that it may be involved in the cytostatic effect of blue light. Moreover, the inhibitory effect was stronger when high irradiation was used (0.04, 0.07, 0.14, 0.22, 0.30, 0.37, 0.45, 0.56, or 1.12 J/cm^2^). The authors found that higher irradiance (0.93 mW/cm^2^) resulted in a greater increase in ROS production and impairment of mitochondrial membrane potential (MMP) than lower irradiance (0.31 mW/cm^2^) [45].

## 7. The Clinical Use of Blue Light

Because of anti-inflammatory and antiproliferative properties of blue light, it may be beneficial for chronic inflammatory skin diseases such as psoriasis vulgaris, atopic dermatitis, and eczema. However, the number of studies assessing the efficacy of blue light treatment in these entities is limited.

### 7.1. Psoriasis

There are six clinical studies assessing the use of blue light in the treatment of psoriasis vulgaris. No major side effects were observed. Only one study did not show the effectiveness of blue light [39,40,46,47,48,49]. Maari et al. treated 17 patients with plaque psoriasis with blue light (λ = 417 nm, 10 J/cm**^2^**, 8.5 mW/cm**^2^**) three times per week for 4 weeks. After 4 weeks, no improvement was observed in the mean psoriasis severity scores of the irradiated plaques [46]. In the prospective, randomized, double-blind study, a statistically significant improvement was demonstrated in the treatment of psoriasis vulgaris using blue light irradiations. Two groups of patients (λ = 420 nm and 453 nm, 90 J/cm**^2^**, 100 mW/cm**^2^**) participated in irradiations each day (15 min) for 4 weeks. The significant improvement of the Local Psoriasis Severity Index (LPSI) was seen in both groups [39]. Probably comparing to the previous study, the better effect was achieved because of the use of higher doses (90 J/cm**^2^** vs. 10 J/cm**^2^**) and the higher number of irradiations (seven per week vs. three per week). In the next prospective, randomized, double-blind study analyzing two groups of patients (low-intensity and high-intensity group), the therapeutic efficacy of blue light (λ = 453 nm, 100 mW/cm**^2^** vs. 200 mW/cm**^2^**) was observed after 12 weeks and compared to the control in both groups. Moreover, in patients receiving a higher intensity of blue light, a significant improvement of LPSI symptoms compared with the control group was demonstrated at each study period. Complete resolution of psoriatic lesions was observed in two patients, which was not achieved in the previous study. This may indicate that longer treatment of blue light is more effective (12 weeks vs. 4 weeks) [47]. Kleinpenning et al. showed that the use of 10% salicylic acid in petrolatum with blue light (λ = 420 nm, 100 mW/cm**^2^**), as well as with red light, caused a statistically significant improvement in the clinical plaque severity scores within 4 weeks of treatment of 20 patients with psoriasis vulgaris. As severe scaling makes light penetration more difficult, salicylic acid was used to remove the plaques. Moreover, there is a hypothesis that, because of the presence of an endogenous photosensitizer-protoporphyrin IX (PPIX) in psoriatic plaques, photodynamic therapy may be a promising therapeutic option even without aminolevulinic acid application [40]. In a recent prospective, randomized study, Krings et al. investigated the effectiveness of blue light (λ = 453 nm, high irradiance level 600 mW/cm**^2^**, 15 min and 30 min) in the treatment of mild psoriasis vulgaris. It was compared to the standard topical calcipotriol, showing similar effectiveness. Both groups (15 min vs. 30 min) achieved a statistically significant improvement of approximately 50% after 12 weeks of treatment preformed each day. Thus, different fluences (38 J/cm^2^ in 15 min and 76 J/cm^2^ in 30 min) caused a similar effect [48]. This indicates that the improvement of the blue light treatment depends not only on the fluence and intensity of irradiations, but also on the total length of treatment and the length of each cycle. Higher fluence, intensity, and longer total treatment time resulted in better outcomes (Table 1). However, in the recent study, the authors demonstrated that extension of duration of irradiation with higher fluencies did not lead to the improvement in effectiveness [48]. The positive effects of blue light were also confirmed in the prospective clinical study of 30 patients with a mild psoriasis vulgaris. The evaluation of the effectiveness of local blue light treatment showed a statistically significant improvement in the mean LPSI and DLQI [49].

### 7.2. Atopic Dermatitis and Eczema

There are only three studies investigating the use of blue light in the treatment of atopic dermatitis and eczema. In a study of 10 patients with atopic dermatitis/hand and foot eczema, focal blue light (40% between 400 and 500 nm, 26% between 400 and 450 nm, 42 J/cm**^2^**, 30 min three times per week for 4 weeks) was shown to cause a significant clinical improvement (Dyshidrosis Area and Severity Index (DASI) before 34.9 vs. after 23.3) compared to the control irradiations (DASI before 32.8 vs. after 34.9) [50]. In another study, 36 adult patients with severe, chronic atopic dermatitis attended full body blue light irradiation (66% between 400 and 500 nm, 28.9 J/cm**^2^**). In total, between two and five cycles (five daily irradiations) were performed over a 6-month period, and between cycles the patients used topical corticosteroids. At 15 days, and at 3 and 6 months after starting the study, a decrease of disease severity (EASI) was observed of 29%, 41%, and 54%, respectively. Reduction in the severity of itching was one of the initial symptoms of improvement that occurred. In addition, the authors showed improvements in sleep quality and quality of life [51]. Additionally, in the prospective, randomized study, it was shown that blue light (λ = 453 nm, 90 J/cm**^2^**) used locally was safe and induced an improvement in eczema lesions [52].

### 7.3. Acne

There is an increasing number of studies demonstrating the beneficial effects of blue light treatment in acne vulgaris [53,54,55,56,57,58,59,60]. The main hypothesis is that the positive effect is related to the reduction of follicular colonization of Propionibacterium acnes, which may be related to the activation of endogenous bacterial porphyrins by blue light [61]. The most important clinical trials are included in Table 2. In the study analyzing the impact of blue light (λ = 415 nm) and red light (λ = 630 nm) on lipid formation, blue light significantly inhibited sebocyte proliferation depending on the dose. This effect was only minimal when analyzing red light. However, based on a lipogenesis study, it was demonstrated that 630 nm light downregulated lipid production. This phenomenon that blue and red light may interfere with lipid generation in sebocytes suggests positive potential in the treatment of acne by inhibiting sebum formation [62].

### 7.4. Photodynamic Therapy (PDT)

A method of treatment that uses visible light, including blue light, is photodynamic therapy. It is mainly used for nonmelanoma skin cancers and actinic keratosis, but the list of indications is enlarging. It is necessary to apply a photosensitizing substance followed by a light irradiation [63,64,65,66,67]. The reaction between the photosensitizer and light leads to the formation of cytotoxic reactive oxygen species (ROS), and as a result, to the destruction of targeted cells [68,69]. The most commonly used topical substances are 5-aminolevulinic acid (5-ALA) and metyl-aminolevulinate (MAL), which are then metabolated to protoporphyrin IX (PPIX) [66]. The spectrum between 400 and 700 nm causes photoexcitation of PPIX and the highest peak is at blue light (λ = 405 nm) [66,70]. However, the penetration of blue light into the skin is more superficial than the penetration of red light [67]. As a consequence, only red light is used to treat skin cancers, whereas blue light may be used for the nonhyperkeratotic actinic keratosis therapy [63,64,71].

## 8. Conclusions

Phototherapy is an important method of dermatological treatments. The main factors responsible for biological changes induced by blue light are light parameters. Developing accurate protocols is crucial, as clinical effects are not only related to the duration of the treatment, but also to the dose and intensity of irradiations. The main factors involved in the response of cells to blue light are NO and ROS. However, the detailed mechanism is still not fully understood. It was demonstrated that blue light induces an anti-inflammatory and antiproliferative effect; thus, it may be beneficial for chronic inflammatory skin diseases such as atopic dermatitis, eczema, and psoriasis. Additionally, the positive effect on the treatment of acne vulgaris and on hair growth was also shown. Another beneficial effect is the reduction of itching, observed in some studies, which may be of great importance in the treatment of patients with skin diseases. On the other hand, there are some studies indicating the negative effects of blue light such as increased oxidative stress, reduced antioxidative capacity of fibroblasts, desynchronization of the cells’ nighttime rhythm, and increased inflammatory response; thus, there are some conflicting results. It seems that this method is safe and the only observed clinical adverse effect may be transient hyperpigmentation. However, there are no studies considering the long-term effects of blue light on the skin.

## Figures and Tables

**Table 1:** Blue light in psoriasis vulgaris, atopic dermatitis, and eczema.

Skin Disease	Treatment Protocol	Irradiation Days Per Week	Irradiation Time (min)	Numer of Patients	Peak Wavelength (nm)	Intensity (mW/cm^2^)—Irradiance	Fluence (J/cm^2^)—Dose	Initial Seveity Index	Outcome	Reference	Year
Psoriasis vulgaris	3 times per week for 4 weeks	3	No information	17	417	8.5	10	LPSI around 6,6	No significant imporvement	[46]	2003
Psorisasis vulgaris	Every day (15 min) for 4 weeks	7	15	40	420 (group 1), 453 (group 2)	100	90	LPSI 5	Significant improvement in both groups	[39]	2011
Psoriais vulgaris	3 (20 min) times per week for 4 weeks	3	20	20	420	100	“high dose”	LPSI 7,7	33,9 % improvement,	[40]	2012
Psoriasis vulgaris	Every day (30 min) for 4 weeks and 3 times per week for the next 8 weeks	7 for 4 weeks and 3 for 8 weeks	30	47	453	100 and 200	90	LPSI 5,17 (200 mW/cm^2^ group) LPASI 5,52 (100 mW/cm^2^ group)	improvement of LPSI in both groups. 29.2% achieved reduction of LPSI between ≥25 and <50%, 33.3% between ≥50 and <75%, and 16.7% of more than 75%	[47]	2015
Psoriasis vulgaris	15 min or 30 min daily for 12 weeks	7	15 or 30	51	453	600	38 and 76	LPSI 5,31 (group 1), LPSI 4,8 (group 2)	improvement of about 50% in both groups	[48]	2019
Atopic dermatitis/hand and foot eczema	30 min 3 times per week for 4 weeks	3	30	10	40% between 400–500, 26% between 400–450	23	42	DASI (dyshidrosis area and severity index) 33,85	Significant improvement (DASI 23,3)	[50]	2005
Atopic dermatitis	1 cycle = 5 consecutive irradiations. 2–5 cycles for maximum 24 weeks	5	24 min of each side of the body	36	66% between 400–500 nm	No information	28,9	EASI 20,6 (6,8–54)	Improvement of about 54%	[51]	2011
Eczema	3 times per week for 4 weeks	3	No information	21	453	No information	90J	Local ESI (local Eczema Severity Index) 4	significant improvement of Local ESI (1,9)	[52]	2016

**Table 2:** Blue light in acne vulgaris.

Skin Disorder	Treatment Protocol	Irradiation Days Per Week	Irradiation Time	Wavelength, Intensity	Number of Patients	Outcome	Reference
Acne vulgaris (mild to moderate, facial, inflammatory)	every other day for 8 weeks, with final assessment 4 weeks post-treatment.	7	No information	414 nm	26 (treatment group), 15 (control group)	Reduction of inflammatory lesion by 50.02%. Maximum effect at week 8–12. After week 12 all patients achieved improvement.	[53]
Acne vulgaris (mild to moderate)	twice a week, with an interval of two days, for 4 weeks	2	20 min	405 +/− 10 nm blue light at the power of 30 mW/cm^2^	10 (blue light), 10 (red light)	Reduction of lesions by 71.4%. The mean number of lesions decreased from 19.2 to 5.5 after 8 irradiations.	[54]
Acne vulgaris	twice a week up to 5 weeks	2	No information	407–420 nm, 90 mW/cm^2^	30	Reduction of lesions by 64%. Within week 5 77% of patients achieved improvement.	[55]
Acne vulgaris	once or twice a week.	1–2	30 min (face), 45 min (back)	405 and 420 nm	10	Improvement was observed in 80% of patients	[56]
Acne vulgaris (mild to moderate facial acne)	twice a week for 4 weeks	2	14 min	415–425 nm (peak 420 nm)	21	Significant reduction of lesions	[57]
Acne vulgaris (facial acne)	Twice a week for 4 weeks	2	15 min	420 nm	28	Lesions improved by 64.7%	[58]
Acne vulgaris (mild-to-moderate inflammatory acne on the face)	Once a day for 8 weeks	7	6 min	414 nm	21	Reduction of open and closed comedomes, papules	[59]
Acne vulgaris (mild to moderate)	Twice a week for 4 weeks	2	10/20 min	409–419 nm at 40 mW/cm^2^	30	Reduction of lesions (at week 8–12)	[60]

## Data Availability

No new data were created or analyzed in this study. Data sharing is not applicable to this article.
